# A role for seed storage proteins in *Arabidopsis* seed longevity

**DOI:** 10.1093/jxb/erv348

**Published:** 2015-07-16

**Authors:** Thu-Phuong Nguyen, Gwendal Cueff, Dwayne D Hegedus, Loïc Rajjou, Leónie Bentsink

**Affiliations:** ^1^Wageningen Seed Lab, Laboratory of Plant Physiology, Wageningen University, 6708 PB Wageningen, The Netherlands; ^2^Department of Molecular Plant Physiology, Utrecht University, 3584 CH Utrecht, The Netherlands; ^3^INRA, Institut Jean-Pierre Bourgin, UMR 1318 INRA-AgroParisTech, ERL CNRS 3559, Laboratory of Excellence ‘Saclay Plant Sciences’ (LabEx SPS), RD10, F-78026 Versailles Cedex, France; ^4^AgroParisTech, Chair of Plant Physiology, 16 rue Claude Bernard, F-75231 Paris Cedex 05, France; ^5^Department of Food and Bioproduct Sciences, University of Saskatchewan, Saskatoon S7N5A9, Canada; ^6^Agriculture and Agri-Food Canada, Saskatoon, Saskatchewan S7N 0X2, Canada

**Keywords:** *Arabidopsis*, carbonylation, proteomics, reactive oxygen species, seed longevity, seed storage proteins.

## Abstract

Proteomics approaches have been a useful tool for determining the biological roles and functions of individual proteins and identifying the molecular mechanisms that govern seed germination, vigour and viability in response to ageing. In this work the dry seed proteome of four *Arabidopsis thaliana* genotypes, that carry introgression fragments at the position of seed longevity quantitative trait loci and as a result display different levels of seed longevity, was investigated. Seeds at two physiological states, after-ripened seeds that had the full germination ability and aged (stored) seeds of which the germination ability was severely reduced, were compared. Aged dry seed proteomes were markedly different from the after-ripened and reflected the seed longevity level of the four genotypes, despite the fact that dry seeds are metabolically quiescent. Results confirmed the role of antioxidant systems, notably vitamin E, and indicated that protection and maintenance of the translation machinery and energy pathways are essential for seed longevity. Moreover, a new role for seed storage proteins (SSPs) was identified in dry seeds during ageing. Cruciferins (CRUs) are the most abundant SSPs in *Arabidopsis* and seeds of a triple mutant for three CRU isoforms (*crua crub cruc*) were more sensitive to artificial ageing and their seed proteins were highly oxidized compared with wild-type seeds. These results confirm that oxidation is involved in seed deterioration and that SSPs buffer the seed from oxidative stress, thus protecting important proteins required for seed germination and seedling formation.

## Highlight

Seed storage proteins protect seed against oxidative stress during seed ageing.

## Introduction

Ecologically, seeds represent a critical stage in the survival of higher plants. Seeds are also important for biodiversity conservation, especially for plants producing orthodox seeds, as they provide desiccation tolerance and permit propagation after long-term dry storage. The germination ability of seeds changes over the seed life span in dry storage conditions. In dormant seeds, the first phase after harvest is reflected by a period in which seeds gradually lose dormancy; during this so called after-ripening (AR) period seeds gain full germination ability. Thereafter, dry seeds slowly deteriorate and lose vigour during storage, which ultimately results in germination failure. It is of both ecological and agronomical relevance to understand the mechanisms governing seed vigour loss during ageing.

Seed longevity is affected by storage conditions, including temperature and humidity (related to seed moisture content). It has been shown that both low temperature and low seed moisture content prolong seed life span during storage (Walters, 1998; Walters *et al.*, 2005). Besides long-term storage (natural ageing), accelerated ageing [controlled deterioration test (CDT), artificial ageing], in which seeds are stored at high temperature and relative humidity, can be used to study seed longevity (Tesnier *et al.*, 2002; Rajjou *et al.*, 2008). It is debated whether accelerated ageing mimics natural ageing (Schwember and Bradford, 2010; Groot *et al.*, 2012). However, for *Arabidopsis* seeds, accelerated ageing largely results in the same regulating factors as those identified for natural ageing (Bentsink *et al.*, 2000; Clerkx *et al.*, 2004*b*). Seed longevity is strongly determined by genetic components. Quantitative trait loci (QTLs) for seed longevity after both natural and artificial ageing were identified in *A. thaliana* (Bentsink *et al.*, 2000; Clerkx *et al.*, 2004*b*; Nguyen *et al.*, 2012), barley (*Hordeum vulgare*) (Nagel *et al.*, 2009), lettuce (*Lactuca sativa*) (Schwember and Bradford, 2010), oilseed rape (Nagel *et al.*, 2011), rice (*Oryza sativa*) (Miura *et al.*, 2002; Sasaki *et al.*, 2005; Zeng *et al.*, 2006; Xue *et al.*, 2008; Hang *et al.*, 2014) and wheat (*Triticum aestivum*) (Landjeva *et al.*, 2009). In addition, several *Arabidopsis* mutants and over-expression lines show altered seed longevity phenotypes. Mutations in seed maturation and dormancy genes, such as *LEAFY COTYLEDON1* and *ABSCISIC ACID INTENSITIVE3* (*ABI3*), lead to dramatic reduction in seed viability (Ooms *et al.*, 1993; Clerkx *et al.*, 2004*a*; Sugliani *et al.*, 2009). Debeaujon *et al.* (2000) showed that testa-defective mutants, including the *transparent testa* (*tt*) and the *aberrant testa shape*, have reduced seed longevity. The non-dormant *dog1* (*delay of germination1*) mutant (Bentsink *et al.*, 2006) and *DOG1*-Cvi (Cape Verde Islands) transformed into L*er* (Landsberg *erecta*) also showed reduced seed longevity phenotypes (Nguyen *et al.*, 2012). Vitamin E, an antioxidant preventing non-enzymatic lipid oxidation, has been proven to promote seed longevity since mutants in vitamin E synthesis genes (*vte1* and *vte2*) showed decreased seed longevity (Sattler *et al.*, 2004). *DNA LIGASE4* and *6* are necessary to maintain genome integrity, as revealed by the high sensitivity of the *lig6* mutant and the *lig6 lig4* double mutant to seed ageing (Waterworth *et al.*, 2010). Tobacco (*Nicotiana tabacum*) seeds over-expressing *HaHSFA9* (a heat shock transcription factor isolated from sunflower *Helianthus annuus*) accumulate elevated levels of heat shock proteins and are more tolerant to CDT (Prieto-Dapena *et al.*, 2006). It has been shown that HaHSFA9 enhances longevity of seeds through functional interaction with a *DROUGHT-RESPONSIVE ELEMENT-BINDING FACTOR2* (Almoguera *et al.*, 2009). Bueso *et al.* (2014) showed that the *Arabidopsis isl1-1D* mutant, over-expressing *ATHB25* (*Homeobox25/Zinc finger protein domain*), exhibited enhanced seed longevity due to the increased expression of (*GIBBERELLIC ACID 3-OXIDASE2*), a GA synthesis gene, and thus elevated GA_1_ and GA_4_ content. The authors suggested a connection between GA and seed longevity through the reinforcement of the seed coat. Over-expression of *PROTEIN-L-ISOASPARTATE METHYLTRANSFERASE1* (*PIMT1*) enhances seed longevity and germination vigour in *Arabidopsis* (Oge *et al.*, 2008). In addition, *Arabidopsis* seeds overexpressing *PIMT1* or *PIMT2* from chickpea (*Cicer arietinum*) were remarkably less sensitive to CDT (Verma *et al.*, 2013). Besides PIMT1, which repairs age-induced damage to aspartyl and asparaginyl residues, METHIONINE SULFOXIDE REDUCTASES (MSRs) also repair damaged proteins at methionine residues. Recently, Chaletain *et al.* (2013) reported that MSR abundance and enzyme activity are strongly linked to seed longevity in *Medicago truncatula* and *Arabidopsis*.

Proteomics approaches have been a useful tool for determining the biological roles and functions of individual proteins and identifying the molecular mechanisms that govern seed germination, vigour and viability in response to ageing (Job *et al.*, 2005; Rajjou et al, 2007; 2008). Post-translational modification (PTM) of proteins in dry seeds plays a central role in dormancy release, metabolism resumption, and ageing processes (Arc *et al.*, 2011). These researchers also demonstrated that the accumulation of oxidized (carbonylated) proteins in dry seed is associated with ageing and might induce loss-of-function of proteins and enzymes. Therefore, detoxification of reactive oxygen species (ROS) that result in oxidative stress and maintenance of redox homeostasis are crucial for seed vigour (Rajjou *et al.*, 2008). Due to their abundance in seeds, seed storage proteins (SSPs) are a primary target for oxidation (Davies, 2005). *Arabidopsis* contains two major SSPs, cruciferins and napins. Cruciferins in *Arabidopsis* are 12S globulins encoded by four paralogous genes AT5G44120 (*CRUA*), AT1G03880 (*CRUB*), AT4G28520 (*CRUC*) and AT1G03890 (*CUPIN*). Napins are referred as 2S albumins and belong to a family with five members. Their abundance in *Arabidopsis* seeds and wide range of PTMs can result in marked changes of SSPs during ageing (Job *et al.*, 2005; Wan *et al.*, 2007). This, together with the fact that SSPs are suggested to contributed to seed germination vigour and support early seedling growth when mobilized upon germination (Muntz *et al.* 2001), might imply a role for SSPs in seed longevity.

Overall seed longevity is a complex trait and a better understanding of the underlying molecular mechanisms is required. In a previous study, we identified 12 *GAAS* (*GERMINATION ABILITY AFTER STORAGE*) loci controlling seed longevity after natural ageing in *Arabidopsis* (Nguyen *et al.*, 2012). To investigate seed longevity mechanisms influenced by the *GAAS* loci, here we performed proteome analyses on dry seeds at two physiological states, after-ripened (AR) and 4-year-old (aged) seeds, from four lines with different seed longevities. T-DNA insertion lines for a subset of protein candidates were investigated for their role in seed longevity after accelerated ageing. These analyses revealed that loss of crucifernins and napins reduced seed longevity and identified a role for cruciferins in buffering oxidation during ageing.

## Materials and methods

### Plant material

**Fig. 1.**
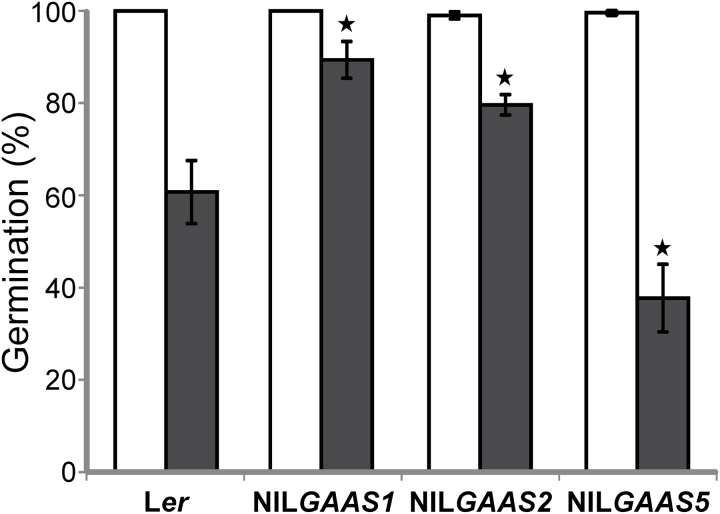
Seed germination after seed dry storage. The germination (%) of L*er* and the near-isogenic lines NIL*GAAS1*, NIL*GAAS2* and NIL*GAAS5* were analysed for after-ripened seeds (open bars) and naturally-aged seeds after four years of storage (filled bars). Averages of four biological replicates with standard errors are presented. The asterisks indicate significant differences between aged NILs and L*er* (*P*<0.05).

The four *A. thaliana* genotypes, namely L*er*, NIL*GAAS1*(-Cape Verde Islands (Cvi)), NIL*GAAS2*(-Antwerp (An-1)) and NIL*GAAS5* (-Shakdara (Sha)), were originally developed as NIL*DOG2*, NIL*DOG22* and NIL*DOG1*, respectively (Bentsink *et al.*, 2010; Nguyen *et al.*, 2012). Those genotypes were grown in a randomized complete block design with replicates in soil as described in Bentsink *et al.* (2010). Seeds of four plants per replicate were bulked. Proteome analyses were conducted for the four genotypes at two physiological stages, fully AR and 4-year-old (aged) seeds. Fully AR seeds are competent to germinate 100% while aged seeds have germination ability reduced; in this study germination phenotype of those seeds were assessed (Fig. 1).

Fully AR seeds were stored in 1.5ml microcentrifuge tubes at −80ºC. Aged seeds came from the same harvest, but were stored in cellophane bags (HERA, papierverarbeitung, Germany) under ambient conditions (∼50% relative humidity and 21°C) for four years. Four biological replicates were used in the proteomic analyses.

The single, double and triple T-DNA insertion lines of cruciferin SSPs and the RNAi line of napin SSP family gene were obtained from Withana-Gamage *et al.* (2013). T-DNA insertion lines (Supplementary Table S1) for candidate genes were screened for homozygous insertions and grown with wild-type Columbia (Col) under greenhouse conditions using rock wool (Grodan, the Netherlands) supplemented with a Hyponex solution 1g l^-1^ (www.hyponex.co.jp), in a randomized complete block design with four replicates per genotype.

### Germination and dormancy assays

Germination assays were performed according to Joosen *et al.* (2010) over a period of 7 d. Briefly, samples of 50−200 seeds each were sown on two layers of blue germination paper (5.6ʹʹ×8ʹʹ Blue Blotter Paper; Anchor Paper Company, http://www.seedpaper.com) equilibrated with 43ml of demineralized water in a plastic tray (15×21cm) (Joosen *et al.*, 2010). Trays were piled and wrapped in a transparent plastic bag and incubated at 22°C under continuous light (30W m^-2^). Pictures of the germination trays were taken twice daily over 7 d. Automatic scoring of germination and statistical analysis were conducted using the Germinator package (Joosen *et al.*, 2010).

Dormancy was calculated as days of seed dry storage required to reach 50% germination as described in He *et al.* (2014).

### Artificial ageing

Artificial ageing was used to evaluate seed longevity of the T-DNA insertion lines. Approximately 200 seeds were placed into an opened 1.5ml eppendorf tube and stored above a saturated NaCl solution in a closed tank that has a ventilator to ensure equal humidity inside the tank monitored by Lascar data logger (80−85% relative humidity and temperature of 40°C) for 0−10 d. After treatment, germination assays were performed as described above.

### Total soluble protein extracts

Thirty milligrams of dry seeds of each sample (four biological replicates) were ground with a mortar and pestle in liquid nitrogen for ∼1min. Extraction buffer and protease inhibitor (Rajjou *et al.* 2008), were added to the seed powder, followed by further grinding for 2min. The solution was placed in 1.5ml microcentrifuge tube and incubated with DNase I 53 units/ml, RNase A 4.9 Kunitz units/ml, and DTT 14mM at 4°C for 1h on rotator (Labinco) at 10rpm. Soluble material extract was collected as supernatant after centrifugation at 14 000rpm at 4°C for 10min.

### 2D gel electrophoresis

Protein separation was performed with 20 µl of protein extract, equivalent to ∼150 µg of protein. 2D gel electrophoresis was conducted as by Rajjou *et al.* (2008, 2011), adapted for gel strips forming an immobilized nonlinear pH gradient from 3 to 11 (Immobilized DryStrip pH 3–11 NL, 24cm; GE Healthcare).

### Comparison of proteome profiles

2D gels were stained with silver nitrate according to Rajjou *et al.* (2008). Stained gels were placed within two layers of cellophane membrane stretched on cassette frames for drying. Images of dry gels were obtained with an Epson Perfection V700 scanner. Quantitative image analysis was conducted using Progenesis Samespot software (v3.2, NonLinear Dynamics) to quantify proteins spots and to detect changes in protein accumulation.

The PCA was performed based on the abundance of the differentially-accumulated protein spots.

Pair-wise statistics were used to detect protein spots that significantly changed in abundance. Two categories were defined; physiological state and genotype and four physiological state comparisons were made between the protein profiles of the two physiological states (aged versus AR) within each genotype. The AR seed proteome profiles of the NILs were also compared to that the Ler genetic background. Protein spots were considered to have been significantly different in abundance if they were higher than or equal to 1.5-fold up or down accumulated and when the *P*-value was equal or smaller than 0.05 according to one-way ANOVA test (Progenesis Samespot software) between the means of the four replicates.

### Protein identification

Due to the high reproducibility of 2D protein patterns, many proteins could be identified based on their position on gel and comparison to reference maps (http://www.seed-proteome.com; Galland *et al.*, 2012). Other proteins spots were excised from the gels, digested with trypsin and identified by LC-MS/MS as described by Arc *et al.* (2012). Peptide sequences were submitted to the XTandem Pipeline (http://pappso.inra.fr/bioinfo/xtandempipeline/) databases to retrieve the full protein sequence and the gene annotation.

### T-DNA insertion genotype analyses

Genomic DNA was isolated from leaves using a modification of the method of Cheung *et al.* (1993). Briefly, 0.5cm diameter leaf sample was ground in 1ml of extraction buffer containing 2M NaCl, 200mM Tris-HCl (pH 8), 70mM EDTA and 20mM Na_2_S_2_O_5_. The grinding was conducted with a stainless steel ball at 30 Hz for 1min in a 96-well plate shaker (Mo Bio Laboratory). Samples were then incubated at 65°C for 1h. Supernatants were collected after centrifugation at 13 000rpm for 10min in 1.5ml eppendorf tubes. DNA was precipitated by adding isopropanol and 10M NH_4_Ac with ratio of 1:0.5:1 to the supernatant. This mixture was incubated at room temperature for 15min and then centrifuged for 20min at 13 000rpm. The DNA pellet was retrieved and rinsed with 1ml of 70% ethanol followed by centrifugation for 5min at 13000rpm to recover the pellet. After drying, the DNA pellet was dissolved in 50 µl distilled water.

Homozygous T-DNA insertion lines were screened with gene-specific primers and T-DNA border-specific primers (Supplementary Table S1). T-DNA plants that amplified only the insertion product were considered to be homozygous mutants.

Polymerase chain reactions (PCR) were performed in a 12.5 µl volume containing ∼30ng DNA, 25 µM of each dNTP, 25ng of forward and reverse primers, 0.05U of DNA polymerase (Firepol, Solis BioDyne), and 312.5 µM of MgCl_2_. The reaction protocol was as follows: denaturation at 95°C for 5min followed by 30 s at 95°C, 30 s annealing at 52 to 57°C (dependent upon the primer pair) and a 45 s to 2min extension (dependent upon the length of the product) at 72°C; this cycle was repeated for 35 times and ended with a final incubation for 10min at 72°C.

The amplified products were separated by agarose gel electrophoresis at concentrations from 1.5 % and higher (w/v) depending on size of differences.

### Detection of protein carbonylation

Carbonylated protein profiles were determined by 1D PAGE of total protein extract followed by derivatization with 2,4-dinitrophenylhydrazine and immunological detection of the DNP adducts with monoclonal anti-DNP antibody (OxyBlot Oxidized Protein Detection Kit; Chemicon) as described previously (Job *et al.*, 2005).

## Results and discussion

### Effect of natural ageing on seed germination ability

The germination ability of AR and 4-year-old (aged) seeds from four genotypes was investigated. The genotypes used are the *Arabidopsis* background line L*er* and three near isogenic lines (NILs) that contain introgression fragments of Cvi, An-1 and Sha accessions, at the position of the earlier identified seed longevity QTL, NIL*GAAS1*-Cvi, NIL*GAAS2*-An-1, and NIL*GAAS5*-Sha, respectively. Upon storage, all genotypes showed a significant reduction in germination percentage, but with a different level of sensitivity to ageing (Fig. 1). NIL*GAAS1* and NIL*GAAS2* had better seed longevity (Gmax=89.3% and 79.6%, respectively) compared with L*er* (Gmax=60.7%), whereas NIL*GAAS5* was less storable (Gmax=37.7%). These results confirmed the seed longevity phenotypes described by Nguyen *et al.* (2012).

### Seed dry storage affects the proteome in a genotype-specific manner

**Fig. 2.**
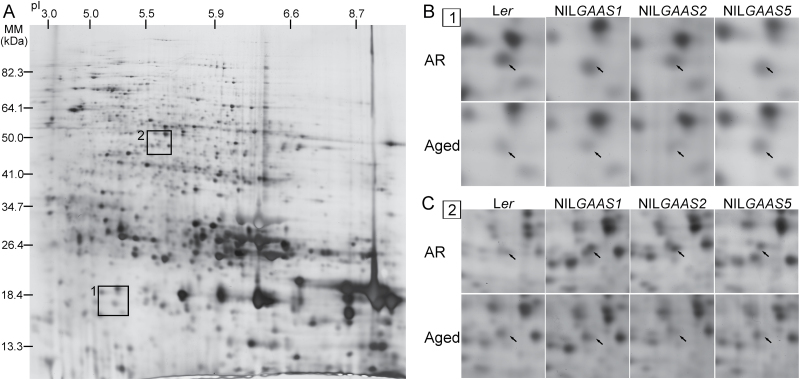
2D gel separation of seed proteins and the effect of ageing on seed protein abundance. (A) 2D gel of total soluble proteins from dry seeds stained with silver nitrate. The areas indicated on the gel (1 and 2) are enlarged in panels B and C. (B) Area 1 selected on the gel depicting the abundance of protein spot ID1345 that contains RPS12C and TPX1 proteins for the four genotypes (L*er* and the near-isogenic lines NIL*GAAS1*, NIL*GAAS2* and NIL*GAAS5*) at two physiological states [after-ripened (AR) and aged]. (C) Area 2 showing the change in abundance of protein spot ID0667, corresponding to VTE1 protein, for the four genotypes at two physiological states. Arrows indicate the position of the proteins.

**Fig. 3.**
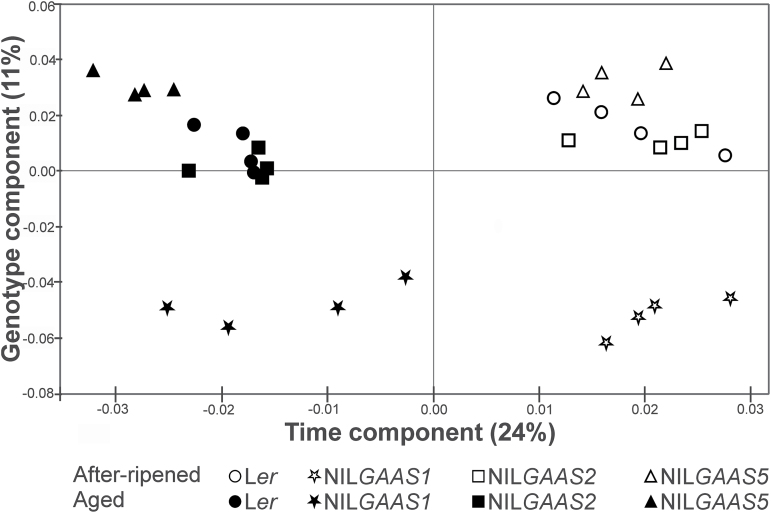
Principal component analysis (PCA) for proteome profiles of L*er* and the near-isogenic lines NIL*GAAS1*, NIL*GAAS2* and NIL*GAAS5*. PCA was performed on the differentially accumulated protein spots in the seven comparisons (*n*=309).

**Fig. 4.**
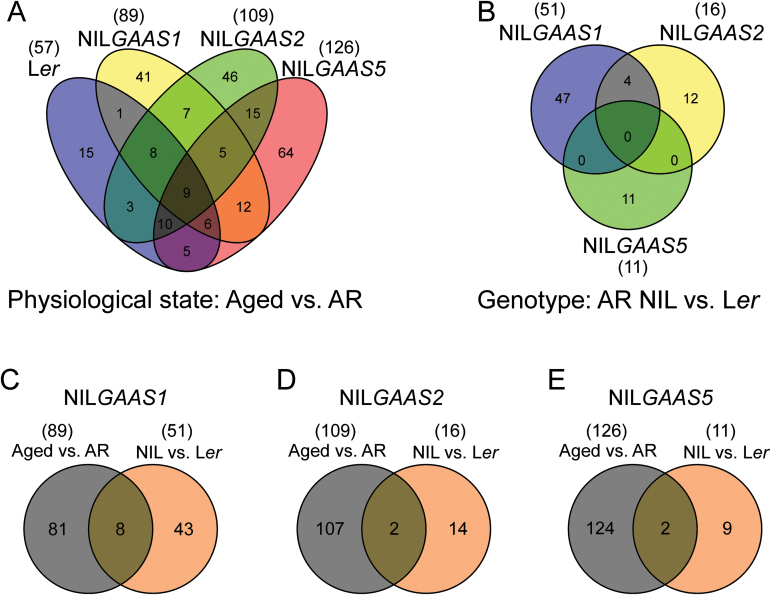
Classification of differentially expressed protein spots. (A) Intersection of proteins that were differentially expressed between the two physiological states, aged versus after-ripened (AR), within Landsberg *erecta* (L*er*) and the near-isogenic lines NIL*GAAS1*, NIL*GAAS2* or NIL*GAAS5* (*n*=247). (B) Intersection of proteins that were differentially expressed between genotypes, each NIL versus L*er* (*n*=74). (C, D, E) Intersection of proteins that were differential expressed in the genotypes NIL*GAAS1*, NIL*GAAS2* and NIL*GAAS5* respectively in the earlier comparisons (as mentioned in A and B).

Proteomic profiling for AR and aged seeds was performed to identify mechanisms and modifications associated with the loss of germination ability during seed dry storage. Total soluble protein extracts were separated using 2D PAGE (Fig. 2A) and seven pair-wise comparisons were made (Supplementary Fig. S1). Pair-wise comparisons between the two physiological states (aged versus AR) for each genotype revealed protein spots that were affected by ageing (57 for L*er*, 89 for NIL*GAAS1*, 109 for NIL*GAAS2*, and 126 for NIL*GAAS5*). The three NILs contain introgression fragments at different genome positions and exhibit different levels of seed longevity. The differences in seed longevity could result from proteome variation already present in the AR seeds, which can be revealed by pair-wise comparisons between the NILs and L*er*. Comparison with the AR L*er* seed proteome allowed the identification of 51, 16 and 11 genotype-specific protein spots for NIL*GAAS1,* NIL*GAAS2* and NIL*GAAS5*, respectively. Some of these protein spots (8, 2 and 2, respectively) overlapped with the aged versus AR comparison (Table 1A). A total of 309 differentially accumulated protein spots were detected in the seven pair-wise comparisons (Supplementary Fig. S1). Principal component analysis (PCA) on the 309 protein spots separated the samples into two groups which represented the two physiological states (AR and aged seeds; Fig. 3). The time component (storage) accounted for 24% of the variation and the genotype component explained 11%. NIL*GAAS1* was the most distinct genotype, and separation of its aged seeds in the PCA might reflect its better longevity on the time component. NIL*GAAS5* is the least storable genotype (Fig. 3). The aged versus AR physiological state comparisons for the four genotypes showed a total of 247 protein spots whose abundance changed significantly (*P*≤0.05) upon ageing (Fig. 4A). A large number of the 247 spots were genotype specific (15 for L*er*, 41 for NIL*GAAS1*, 46 for NIL*GAAS2*, and 64 for NIL*GAAS5*) (Fig. 4A). The three genotype comparisons led to the identification of 74 altered protein spots (Fig. 4B), of which most were unique to the genotypes (47 for NIL*GAAS1*, 12 for NIL*GAAS2*, and 11 for NIL*GAAS5*) (Fig. 4B). The four spots in common between NIL*GAAS1* and NIL*GAAS2* (Fig. 4B; Supplementary Table S2) when compared with L*er* at the AR state might play a role in seed longevity, because both genotypes are more storable than L*er*.

**Table 1.** Genotype specific protein spots that were identified in (A) both physiological state and genotype comparisons and (B) in either physiological state or genotype comparison for near-isogenic lines NILGAAS1, NILGAAS2 and NILGAAS5 (Fig. 4C−E) The table displays protein spots based on the seven comparisons. Spot ID, the gene corresponding to the protein underlying the spot, molecular weight (MW in kD) and the theoretical (Th) and experimental (Exp) isoelectric point (pI), are presented respectively. Furthermore the relative abundance (fold change) of the spots in both types of comparisons (Fig. 1; physiological state and genotype) is indicated. Positive fold changes indicate higher abundances, and negative lower abundances. Spots in bold exhibit seed longevity up or seed longevity down protein profile. Fold changes in bold indicate statistically significant changes. N*G1*, NIL*GAAS1;* N*G2*, NIL*GAAS2*; N*G5*, NIL*GAAS5*. Spots that were identified based on comparison to the reference protein map (http://www.seed-proteome.com) are labelled with ^R^. n.i., not identified.

**A**	Spot ID	Gene	Protein	MW (kDa)		pI		Relative abundance (fold change)						
				Th	Exp	Th	Exp	Physiological state: Aged vs. AR				Genotype AR: NIL vs. L*er*		
								L*er*	N*G1*	N*G2*	N*G5*	N*G1*	N*G2*	N*G5*
	NIL*GAAS1*													
	**ID1279**	n.i.	n.i.	n.i.	22.82	n.i.	8.68	-1.0	**2.5**	-1.1	1.5	**-2.6**	1.3	-1.3
	**eID0255** ^R^	AT1G03890	Cupin family protein	49.67	29.33	5.45	5.63	-1.0	**1.9**	1.0	1.0	**-2.3**	-1.0	-1.0
	**ID0426**	n.i.	n.i.	n.i.	65.51	n.i.	5.41	1.2	**1.8**	1.2	1.4	**-1.7**	-1.4	-1.2
	ID1104	AT1G03880	Cruciferin B	50.56	27.38	7.0	5.61	1.1	**1.5**	1.0	1.1	**1.9**	1.1	1.0
		AT5F35590	20S proteasome alpha subunit	27.29		5.66								
		AT1G03890	Cupin family protein	49.67		5.45								
	eID0138 ^R^	AT4G28520	Cruciferin C	58.24	25.40	6.99	5.81	**1.9**	**1.5**	**1.5**	**1.6**	**2.3**	1.3	1.0
	ID0994	AT1G54870	Oxidoreductase family protein	36.76	32.75	8.76	5.65	**2.3**	**1.7**	**2.0**	**2.4**	**1.5**	1.2	1.3
		AT4G28520	Cruciferin C	58.24		6.99								
	**ID0715**	AT1G03880	Cruciferin B	50.56	47.74	7.00	6.02	-1.2	**-1.6**	-1.3	**-1.6**	**1.6**	-1.0	1.1
		AT1G74960	Fatty acid biosynthesis 1	57.60		7.93								
	**ID0537**	AT2G14170	Aldehyde dehydrogenase 6B2	53.40	58.77	5.79	5.68	**-2.0**	**-3.1**	**-2.3**	**-2.0**	**1.5**	1.3	1.1
		AT5G08670	ATP synthase beta chain 1	59.63		6.53								
		AT5G08680	ATP synthase beta chain	59.86		6.45								
		AT5G08690	ATP synthase beta chain 2	59.71		6.60								
	NIL*GAAS2*													
	**ID0458**	AT3G20050	T-complex protein 1 alpha subunit	59.23	63.81	6.22	5.87	-1.2	1.1	**1.5**	1.1	-1.4	**-2.0**	-1.2
	**ID0955**	n.i.	n.i.	n.i.	38.44	n.i.	7.29	1.4	**-1.7**	**-2.1**	**-1.8**	1.4	**1.7**	1.5
	NIL*GAAS5*													
	eID0228	AT5G19510	Elongation factor EF1B	24.20	43.24	4.17	3.88	-1.6	1.3	1.2	**1.7**	-1.3	-1.1	**-1.6**
	ID1146	AT2G31670	Unknown protein	28.86	25.36	6.96	5.10	1.1	1.3	1.4	**1.8**	-1.0	-1.3	**-1.5**

B	Spot ID	Gene	Protein	MW (kD)		pI		Relative abundance (fold change)						
				Th	Exp	Th	Exp	Physiological state: Aged vs. AR				Genotype AR: NIL vs. L*er*		
								L*er*	N*G1*	N*G2*	N*G5*	N*G1*	N*G2*	N*G5*
	NIL*GAAS1*													
	ID0765	AT3G52880	Monodehydroascorbate reductase 1	50.16	43.31	8.38	6.08	-1.3	-1.3	-1.4	1.3	**2.6**	-1.0	-1.6
	ID0712	AT5G28840	GDP-D-mannose 3ʹ,5ʹ-epimerase	42.76	45.14	6.15	5.78	1.2	1.2	-1.0	-1.0	**1.9**	-1.1	1.0
	ID0734	AT5G28840	GDP-D-mannose 3ʹ,5ʹ-epimerase	42.76	44.99	6.15	5.74	1.2	**1.7**	1.2	1.5	1.1	-1.1	-1.1
	ID0951	AT1G18080	Receptor for activated C kinase 1A	35.75	38.56	7.81	7.13	-1.1	-1.6	-1.3	-1.8	**1.6**	1.4	1.4
	NIL*GAAS2*													
	ID0206	AT5G52300	Responsive to dehydration 29B	65.97	94.21	4.81	5.05	-1.5	-1.2	**-1.7**	-1.5	-1.3	-1.2	1.1
	ID0196 ^R^	AT5G52300	Responsive to dehydration 29B	65.97	94.21	4.81	5.00	-1.4	-1.9	1.0	1.1	1.2	**-1.6**	-1.5
	ID0762	AT2G15430	RNA polymerase II	35.46	42.82	4.39	4.46	-1.3	-1.1	**-1.6**	-1.1	-1.4	-1.1	-1.3
	NIL*GAAS5*													
	ID1505	AT4G27160	Napin AT2S3	18.76	9.85	7.87	8.69	1.5	1.1	-1.2	**1.7**	-1.0	1.2	1.2
	ID0632	AT5G38470	Radiation sensitive 23D	40.07	50.00	4.29	4.60	1.1	-1.1	-1.0	-1.1	1.0	-1.1	**-2.0**
	ID0258	AT3G15670	Late embryogenesis abundant protein	24.19	26.57	9.43	8.95	-1.2	-1.0	1.1	1.1	1.0	-1.1	**-1.9**
	ID1144	AT3G15670	Late embryogenesis abundant protein	24.19	29.73	9.43	7.79	-1.1	-1.2	-1.1	**-1.5**	1.0	1.0	1.2
	ID0976	AT3G17520	Late embryogenesis abundant protein	32.56	36.11	5.02	5.54	1.2	1.2	1.4	**1.6**	1.0	-1.1	-1.2
	ID0448	AT2G19900	NADP-dependent malic enzyme 1	64.28	n.i.	6.73	n.i.	-1.3	-1.7	-1.7	**-1.7**	1.1	-1.1	-1.0

Genotype-specific protein spots include those differentially expressed in only one genotype derived from physiological comparisons (aged versus AR seeds; Fig 4A), and those identified in genotype comparisons (AR seeds of NILs versus L*er*; Fig 4B). Overlapping spots of both comparison types are presented in Fig. 4C−E and Table 1A (8 for NIL*GAAS1*, 2 for NIL*GAAS2* and 2 for NIL*GAAS5*). Generally, these results indicate that seed dry storage affects the proteome in a genotype-specific manner. Furthermore, since the NILs used in this study possess different introgressed genomic regions at seed longevity QTLs (Nguyen *et al.*, 2012), several distinct pathways might be involved in seed ageing which could be manifested as unique elements in their respective seed proteomes. These differences can either be causes or consequences of ageing and one cannot exclude the differences in the AR seed proteomes are caused by the nature of the introgression in the NILs and are therefore unrelated to seed longevity.

### Diverse genetic pathway involved in seed longevity of the different genotypes

To identify the factors underlying the protein differences both reference mapping and mass spectrometry (MS) analysis was performed. Approximately 70% of the differentially accumulated protein spots could be identified. Seed proteins are targets of various modifications (reviewed by Arc *et al.*, 2011), including carbonylation and S-nitrosylation, especially in dry storage. As a consequence of these modifications, proteins can be represented by several isoforms with different molecular weight (MW) and/or isoelectric point (pI). Thus multiple spots of the same protein were identified.

Investigation of the identified proteins in the four genotypes, L*er*, NIL*GAAS1*, NIL*GAAS2* and NIL*GAAS5* revealed that possible diverse genetic pathways are involved in seed longevity which is reflected by proteins for which the isoforms are significantly altered in abundance in a genotype-specific manner.

### Mechanism related to seed ageing in NIL*GAAS1*

NIL*GAAS1* is the most storable line of the four tested genotypes (Fig. 1). NIL*GAAS1* has 41 unique protein spots that accumulate differently in aged compared to AR seeds (Fig. 4A), and 47 when comparing AR seeds of NIL*GAAS1* to that of L*er* (Fig. 4B). Eight spots were at the intersect of both comparison types for NIL*GAAS1* (Fig. 4C; Table 1A), of which two, ID0715 and ID1104, contained CRUB. NIL*GAAS1* carries the truncated *CRUB* Cvi allele (Hou *et al.*, 2005) resulting in a CRUB α-subunit with a lower MW compared to that from the L*er* allele, which might account for the identification of CRUB in NIL*GAAS1*.

Dry seeds are well equipped to confront oxidative stress during storage due to their low water content and reduced metabolic activity. Auto-oxidation leading to accumulation of ROS occurs in dry seed where proteins are the major targets of oxidative damage because of their abundance and high affinity with radicals (Davies, 2005). To control ROS-induced damage, seeds have detoxification mechanisms to scavenge or inactivate ROS. We found two proteins that are involved in the ascorbate antioxidant metabolism pathway in NIL*GAAS1*. Protein spot ID0765 (Table 1B) corresponds to MONODEHYDROASCOBATE REDUCTASE1 (MDAR1), a well known antioxidant enzyme that removes hydrogen peroxide at the ascorbate-glutathione cycle (Bailly, 2004). T-DNA insertion mutant analysis for seed longevity showed that *mdar1.1* and *mdar1.2* did not differ in seed longevity compared to Col (Supplementary Fig. S2). The lack of a phenotype might be due to redundancy since the MDAR gene family contains five members. Over-expression of *AtMDAR1* in tobacco conferred enhanced tolerance to ozone, salt and osmotic stresses (Eltayeb *et al.*, 2007) suggesting that the ascorbate-glutathione cycle may play a role in seed ageing. GDP-D-MANNOSE 3′,5′-EPIMERASE (GME) is a key enzyme involved in ascorbate (vitamin C) synthesis in plants (Wolucka *et al.*, 2001; Wolucka and Van Montagu, 2003). NIL*GAAS1* AR seeds had higher levels of GME (ID0712), which likely explains the higher resistance to ageing of NIL*GAAS1* compared to L*er* seeds (Table 1B). The GME isoform ID0734 was found more abundant in aged than AR seeds, which could indicate GME modification during ageing. Homozygous *GME* T-DNA insertion lines could not be isolated indicating that GME is an essential protein (Supplementary Table S1).

RECEPTOR FOR ACTIVATED C KINASE1 (RACK1) isoform ID0951 was more abundant in AR seeds of NIL*GAAS1* than that of L*er* (Table 1B), which might also contribute to the better seed longevity of this NIL. RACK1 is a multi-function protein that plays a regulatory role in diverse signal transduction pathways and its transcripts are present during seed germination (Chen *et al.*, 2006; Guo *et al.*, 2009). In *Arabidopsis*, the translation initiation factor eIF6-2 interacts with RACK1, a negative regulator of ABA response and positive regulator of GA signalling (Guo *et al.*, 2011; Fennell *et al.*, 2012). It was demonstrated that ABA inhibited RACK1 and eIF6 gene expression (Guo *et al.*, 2011). Thus seed germination of a *rack1A* T-DNA insertion line was examined, however, did not significantly differ from that of wild-type Col (Supplementary Fig. S2).

### Mechanism involved in seed ageing expressed in NIL*GAAS2*

NIL*GAAS2*, the second most storable genotype, had 46 unique protein spots that differentially accumulated when aged and AR seeds were compared (Fig. 4A) and 12 when AR seeds of NIL*GAAS2* were compared to that of L*er* (Fig. 4B). Two spots were common in both comparisons (Fig. 4D; Table 1A). Although no specific pathway was identified, there are interesting proteins that might be involved in seed longevity in this NIL.

Spot ID0458 (Table 1A) corresponds to the T-complex Protein 1 α Subunit (TCP1). Several members of the TCP1 protein family were described to be up-accumulated during *Arabidopsis* seed dormancy release, suggesting that they could play a central role in seed germination (Arc *et al.*, 2012). RESPONSIVE TO DEHYDRATION29B (RD29B) was identified in two protein spots, ID0206 and ID0196 (Table 1B). The increased level of RD29B could be a marker for increased seed longevity.

It was noted that *de novo* transcription is not required for germination since seeds are able to germinate until radical protrusion in the presence of α-amanitin (a transcription inhibitor targeting RNA POLYMERASE II); however subsequent seedling growth was prevented (Rajjou *et al.*, 2004). Newly synthesized transcripts might also be necessary for germination of aged seed. The low abundance of RNA POLYMERASE II (AT2G15430, ID0762), 1.6-fold less abundant after ageing of NIL*GAAS2* seeds (Table 1B), may contribute to reduced seed germination after storage. Homozygous T-DNA insertion lines for RNA POLYMERASE II could not be isolated (Supplementary Table S1).

### Seed longevity mechanisms in NIL*GAAS5*

NIL*GAAS5* is the most sensitive genotype to ageing in this study (Fig. 1). The reduced seed longevity in NIL*GAAS5* is caused by the *DOG1-Cvi* allele, as was revealed by complementation cloning (Nguyen *et al.*, 2012). DOG1 protein accumulates during seed maturation and remains stable throughout seed storage, however it is modified during after-ripening (Nakabayashi *et al.*, 2012). We did not identify DOG1, likely because it is rather stable and not modified anymore at later stages during dry storage. NIL*GAAS5* had 64 unique protein spots that differentially accumulated when aged and AR seeds were compared (Fig. 4A), and 11 when AR seeds of NIL*GAAS5* were compared to that of L*er* (Fig. 4B). Two spots, eID0228 and ID1146, encoding elongation factor EF1B and an unknown protein (Fig. 4E; Table 1A) were common to both comparisons.

One of the proteins differentially accumulating in NIL*GAAS5* is RADIATION SENSITIVE23D (RAD23D), its protein spot (ID0632) is down regulated compared to L*er* (Table 1B). RAD23D was suggested to participate in DNA damage repair since the two carrot *(Daucus carota*) RAD23 isoforms rescue the UV-sensitive phenotype of the *rad23* deletion mutant in yeast (*Saccharomyces cerevisiae*) (Sturm and Lienhard, 1998). During storage seeds are subjected to DNA damage and genome instability, which are considered to be a main cause of reduced germination after ageing. The maintenance of a functional DNA repair complex is essential for long-term survival (reviewed by Rajjou *et al.*, 2008). However, since *RAD23D* is located in the introgression region of NIL*GAAS5*, we cannot exclude the possibility that it affects seed longevity independently from *DOG1*.

Another group of differentially accumulating proteins are the identified LATE EMBRYOGENESIS ABUNDANT FAMILY-4 PROTEINS; AT3G17520 in spot ID0976 and AT3G15670 in spot ID0258 and ID1144 (Table 1B). The association of LEA AT3G15670 and seed germination after ageing was examined by T-DNA insertion mutant analysis (Supplementary Table S1); however, the *lea* mutant exhibited similar seed longevity compared to Col (Supplementary Fig. S2). NADP-DEPENDENT MALIC ENZYME1 (NADP-ME1) in spot ID0448 (Table 1B) was lower abundant in aged compared with AR seeds of NIL*GAAS5*, which makes it a possible marker for seed longevity.

**Fig. 5.**
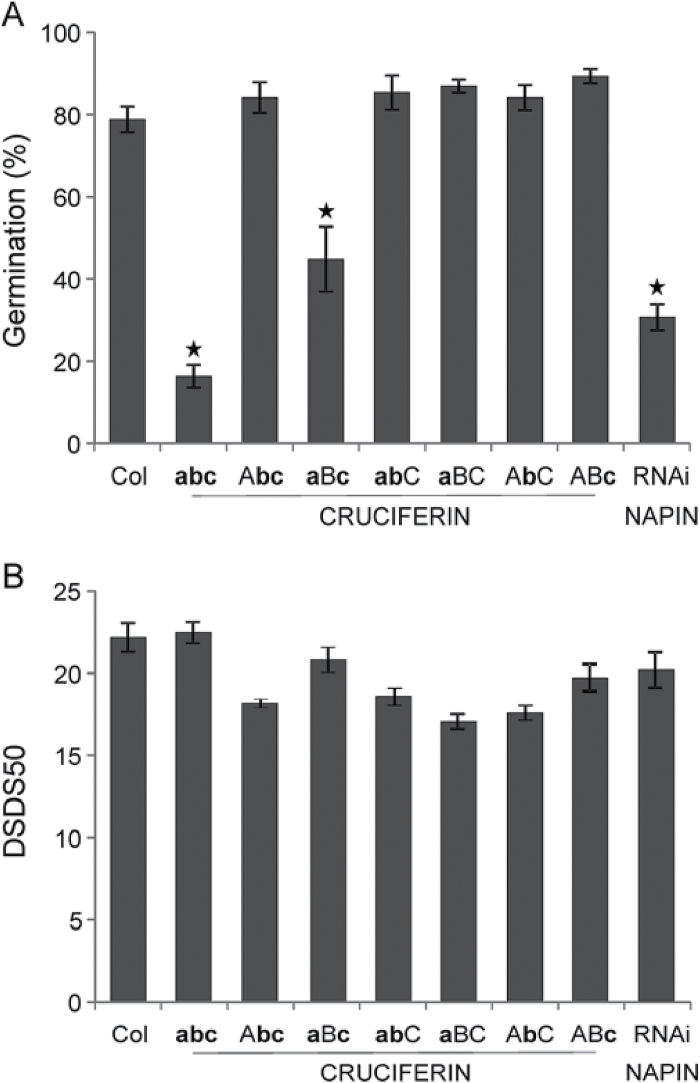
The effect of seed storage proteins (SSPs) on seed longevity and seed dormancy.(A) Seed longevity presented as germination (%) of different SSP knock-out lines was measured after 10 d of artificial ageing. The lines include the wild-type Col, as well as single (*crua*, **a**BC; *crub*, A**b**C and *cruc*, AB**c**), double (*crub cruc*, A**bc**; *crua cruc*, **a**B**c** and *crua crub*, **ab**C) and triple (*crua crub cruc*, **abc**) knock-out lines of cruciferins, and an RNAi napin line that is depleted of napins. (B) Seed dormancy presented as days of seed dry storage required to reach 50% germination (DSDS50) of Col and different SSP knock-out lines.

AT2S3, one of the five 2S albumin or napin isoforms (Krebbers *et al.*, 1988; Van der Klei *et al.*, 1993), was more abundant in aged NIL*GAAS5* (the most sensitive genotype to ageing) (Table 1B). The AT2S3 protein spot ID1505 might be a degradation product due to its altered MW and pI (Table 1B). To examine if napins could affect seed longevity, a napin RNAi line was analysed for seed longevity (Withana-Gamage *et al.*, 2013) (Supplementary Table S3). The line, which is depleted of napins and has a reduced protein content mainly in the endosperm, was more sensitive to ageing than wild-type Col (Fig. 5).

### Pathways/proteins that are generally affected during storage

#### Energy metabolism and seed longevity

Carbohydrate metabolism is important for germinating seeds since it provides energy and intermediate metabolites for seed germination and seedling establishment. The metabolic pathways related to energy production are glycolysis, the oxidative pentose phosphate pathway (OPP), fermentation, the tricarboxylic acid cycle (TCA), the glyoxylate cycle, and the electron transport chain (Supplementary Fig. S3). The abundance of several protein isoforms that encode for enzymes in these pathways were altered in the seed proteome upon storage (Table 2). Similar changes in the seed proteome upon ageing has also been found previously (Rajjou *et al.*, 2008). In order to investigate the importance of these enzymes for seed longevity we have investigated T-DNA insertion lines of these genes for their seed longevity behaviour. The list of T-DNA insertion mutants that was tested is provided in Supplementary Table S1. For none of the mutants investigated seed longevity phenotypes have been revealed. It is possible that these enzymes are not important for seed longevity however we expect that the lack of phenotypes are caused by gene redundancy. For example, for the glycolysis enzyme UDP GLUCOSE URIDYLYLTRANSFERASE (UGP) we could only obtain one homozygous mutant (*ugp1*) and this mutant did not show a seed longevity phenotype (Supplementary Fig. S2), however there are two UGPs in *Arabidopsis* and these likely act redundantly.

**Table 2.** Differentially expressed protein spots upon ageing that are related to translation, energy metabolism, reactivation of cell activity, redox homeostasis, and ABA signalling The table displays protein spots based on the seven comparisons. Spot ID, the gene corresponding to the protein underlying the spot, molecular weight (MW in kD) and the theoretical (Th) and experimental (Exp) isoelectric point (pI), are presented respectively. Furthermore the relative abundance (fold change) of the spots in both types of comparisons (Fig. 1; physiological state and genotype) is indicated. Positive fold changes indicate higher abundances, and negative lower abundances. Fold changes in bold indicate the statistically significant changes. N*G1,* NIL*GAAS1*; N*G2,* NIL*GAAS2*; N*G5,* NIL*GAAS5*. Spots that were identified based on comparison to the reference protein map (http://www.seed-proteome.com) are labelled with ^R^. n.i., not available.

Spot ID	Gene	Protein	MW (kD)		pI		Relative abundance (fold change)						
			Th	Exp	Th	Exp	Physiological state: Aged vs. AR				Genotype AR: NIL vs. L*er*		
							L*er*	N*G1*	N*G2*	N*G5*	N*G1*	N*G2*	N*G5*
Translation													
ID1345	AT2G32060	Ribosomal protein 40S subunit	15.33	16.25	5.70	5.07	**-1.7**	**-2.5**	**-1.5**	1.1	-1.1	-1.3	-1.5
ID0667 ^R^	AT1G57720	Elongation factor EF1Bγ	46.40	49.95	5.40	5.54	-1.4	-1.1	**-1.5**	**-1.5**	1.2	1.4	1.2
eID0228 ^R^	AT5G19510	Elongation factor EF1B	24.20	43.24	4.17	3.88	-1.6	1.3	1.2	**1.7**	-1.3	-1.1	**-1.6**
ID0885	AT1G30230	Elongation factor EF1β	28.77	38.67	4.36	3.98	-1.3	-1.0	**-1.5**	-1.7	-1.2	1.1	1.2
ID1226	AT1G07930	Elongation factor EF1α	49.50	25.11	9.64	7.81	-1.2	-1.6	1.0	-1.1	1.4	1.1	**-2.1**
	AT5G60390	Elongation factor EF1α	49.50		9.64								
Energy metabolism													
ID0621	AT5G17310	UGP-1 glucose uridylytransferase 2	51.92	55.53	5.80	5.80	1.3	1.5	**1.5**	1.3	-1.1	-1.0	-1.1
ID0603	AT3G03250	UGP-1 glucose uridylytransferase 1	51.74	56.94	5.98	5.71	1.1	-1.1	-1.4	**-1.5**	1.3	1.4	1.3
	AT5G17310	UGP-1 glucose uridylytransferase 2	51.92		5.80								
ID0832	AT1G13440	Glyceraldehyde-3-P dehydrogenase C2	36.91	43.15	7.22	6.05	**1.9**	**1.7**	**1.7**	1.3	-1.0	1.2	1.4
	AT3G04120	Glyceraldehyde-3-P dehydrogenase C1	36.91		7.15								
ID0997	AT1G13440	Glyceraldehyde-3-P dehydrogenase C2	36.91	33.67	7.22	5.72	1.7	1.7	**1.9**	**2.7**	1.2	1.1	-1.1
	AT3G04120	Glyceraldehyde-3-P dehydrogenase C1	36.91		7.15								
eID0214	AT2G45290	Transketolase	79.92	81.80	6.55	5.77	**-2.0**	**-1.8**	**-2.3**	-1.8	-1.0	-1.1	-1.1
eID0096	AT3G60750	Transketolase	79.97	78.47	6.32	5.45	**-1.6**	**-1.9**	**-1.6**	**-1.9**	1.2	1.1	1.2
eID0275	AT5G54960	Pyruvate decarboxylase 2	65.82	64.35	5.84	5.54	-1.7	**-1.5**	**-1.7**	**-2.0**	1.2	1.4	1.2
ID1180	AT1G04410	Malate dehydrogenase 1	35.57	25.20	6.51	5.64	1.6	-1.0	1.5	**2.0**	1.3	-1.2	-1.2
ID0448	AT2G19900	NADP-dependent malic enzyme 1	64.28	n.i.	6.73	n.i.	-1.3	-1.7	-1.7	**-1.7**	1.1	-1.1	-1.0
ID0526	AT5G08670	ATP synthase β chain 1	59.63	59.34	6.53	5.35	1.2	1.3	1.2	**1.6**	1.0	1.0	-1.1
	AT5G08680	ATP synthase β chain	59.86		6.45								
	AT5G08690	ATP synthase β chain 2	59.71		6.60								
ID0535	AT5G08680	ATP synthase β chain	59.86	58.73	6.45	5.49	-1.3	**-1.5**	1.2	1.3	1.0	-1.3	-1.4
ID0538	AT5G08670	ATP synthase β chain 1	59.63	58.22	6.53	5.51	-1.3	-1.3	**-1.5**	-1.4	1.1	1.3	1.1
ID0556	AT5G08670	ATP synthase β chain 1	59.63	57.44	6.53	5.49	1.0	**-1.6**	1.1	1.0	1.9	1.4	1.4
Reactivation of cell													
ID0693	AT2G36880	Methionine adenosyltransferase 3	42.50	46.03	6.09	5.73	-1.1	**1.6**	-1.3	-1.4	1.3	1.1	1.2
ID0694 ^R^	AT4G01850	Methionine adenosyltransferase 2	43.25	47.58	5.94	5.65	-1.1	-1.0	**-1.6**	-1.1	-1.0	1.3	1.3
Redox homeostasis													
ID1345	AT1G65980	Thioredoxin-dependent peroxidase 1	17.43	16.25	5.00	5.06	**-1.7**	**-2.5**	**-1.5**	1.1	-1.1	-1.3	-1.5
ID0667	AT4G32770	Vitamin E deficient 1	54.72	49.95	6.26	5.54	-1.4	-1.1	**-1.5**	**-1.5**	1.2	1.4	1.2
ABA signalling													
eID0200	AT1G43890	Responsive to abscisic acid 18	23.53	18.53	5.43	5.79	1.2	1.4	**1.7**	**1.7**	-1.1	-1.4	-1.3

#### The effect of ageing on translation capacity and protein metabolism

Protein translation is essential for seed germination, since the presence of the translation inhibitor cycloheximide prevented radicle protrusion (Rajjou *et al.*, 2004). Furthermore, aged seeds were strongly affected in their translation capacity (Rajjou *et al.*, 2008). This research also demonstrated that many proteins involved in protein metabolism were highly carbonylated in deteriorated seeds. Consistent with previous studies, we observed that the levels of elongation factor EF1B family proteins were lower in aged compared to AR seeds (Table 2). Moreover, spot ID1345, corresponding to the ribosomal protein 40S subunit RPS12C, was reduced in abundance after storage of L*er*, NIL*GAAS1* and NIL*GAAS2* seeds (Fig. 2B; Table 2).

#### Reactivation of cellular activity

Changes in the abundance of METHIONINE ADENOSYLTRANSFERASE (MAT) were observed in both NIL*GAAS1* (MAT3, ID0693; Table 2) and NIL*GAAS2* (MAT2, ID0694; Table 2) after ageing. MAT participates in *S*-adenosylmethionine (Ado-Met) biosynthesis and is important for reactivating cellular activity in germinating seeds (Ravanel *et al.*, 1998; Gallardo *et al.*, 2002). This indicates genotype-specific differences in the dependency on pathways related to Ado-Met metabolism in seed longevity.

#### Redox homeostasis and antioxidants in seed longevity

Seed storage and germination are coupled to extensive changes in the redox state of seed proteins and even in the dry state seed proteins are subjected to various types of PTMs which include redox modifications (Arc *et al.*, 2011; Rajjou *et al.*, 2012). Thioredoxin has a vital role in redox processes, in which it transforms essential proteins from the oxidized to the reduced form and in the process retrieves the molecular function of those proteins. THIOREDOXIN-DEPENDENT PEROXIDASE1 (TPX1) (ID1345) abundance declined in aged compared to AR seeds of L*er*, NIL*GAAS1*, NIL*GAAS2* (Fig. 2B; Table 2). TPX1 is a thioredoxin-dependent peroxidases type II B (Prx IIB), one of the six isoforms in this family, which has a wide range of redox buffering activities (Rouhier and Jacquot, 2005). Thus, TPX1 might be very important for retaining the redox balance during ageing and germination. To investigate the role of TPX1, seed longevity for the *tpx1* mutant was analysed; however, it was similar to the wild-type Col accession (Supplementary Fig. S2). The lack of a visible phenotype for *tpx1* could be due to the nature of the T-DNA insertion (3ʹ-UTR region of the gene) (Supplementary Table S1) or due to redundancy of enzymatic antioxidant systems in seeds, so that missing one might not have obvious effects.

Vitamin E is another antioxidant important for seed longevity since the *vitamin E deficient1* (*vte1*) mutant seeds were more sensitive to artificial ageing than those of the wild type (Sattler *et al.*, 2004). In this study, VTE1 (ID0667) abundance was lower in aged seeds of NIL*GAAS2* and NIL*GAAS5* (Fig. 2C; Table 2) than AR seeds, which is in agreement with previous studies suggesting a role of *VTE1* in seed longevity.

#### The role of seed storage proteins in ageing

Many of the identified protein spots were SSP 12S globulin fragments, a predominant type of SSP referred to as cruciferin (Pang *et al.*, 1988). A set of single, double and triple knock-out lines for *CRUA*, *CRUB* and *CRUC* (Withana-Gamage *et al.*, 2013) (Supplementary Table S3) was analysed to study the role of cruciferins in seed longevity under artificial ageing. Cruciferin single knock-out mutants lacking one of the cruciferin isoforms did not differ in seed longevity compared to wild-type Col (Fig. 5). The *crua cruc* double mutant lacking both CRUA and CRUC exhibited reduced seed longevity, whereas seed longevity for the *crua crub* and *crub cruc* double mutants was unaffected. *CRUB* is poorly transcribed and CRUB is the least abundant cruciferin isoform (Withana-Gamage *et al.*, 2013), thus the *crua cruc* double mutant has very low levels of cruciferin. This explains why double mutants that have eliminated CRUB, but retained the more abundant CRUA or CRUC isoforms, did not show reduced seed longevity. The effect of cruciferin content on seed longevity was even more apparent in the *crua crub cruc* triple mutant which was very sensitive to artificial ageing (Fig. 5A). The role of cruciferins on seed longevity cannot be explained by reduced protein levels since the *crua cruc* double mutant has wild type protein levels in both the endosperm and the embryo (Withana-Gamage *et al.*, 2013). This phenomenon is referred to as seed proteome rebalancing and involves a general increase in the production of other seed proteins to compensate for the loss of a major SSP (Herman, 2014). Defects in seed development that lead to reduced seed longevity often result in reduced seed dormancy levels as well, examples are the *abi3-5*, *dog1-1* and *tt* mutants (Ooms *et al.*, 1993; Debeaujon *et al.*, 2000; Clerkx *et al.*, 2004*a*; Sugliani *et al.*, 2009). However, despite the reduction in SSPs and reduced seed longevity, seed dormancy as measured by days of seed dry storage required to reach 50% germination was unaffected in the *cru* mutants (Fig. 5B). Thus, the loss of germination in the mutant is not caused by the lack of protein reserves. It is possible that the localization and distribution of the proteins is important since the *crua crub cruc* triple mutant contains very small protein storage vesicles with very little protein within (Withana-Gamage *et al.*, 2013). The influences of SSPs can also be due to their modification because SSPs are subject to a wide range of PTMs (Job *et al.*, 2005; Wan *et al.*, 2007), suggesting that the effects of PTMs on SSPs play a role in seed longevity.

### SSPs function as oxidation buffers in seed longevity

Although several T-DNA mutants were tested for seed longevity, SSP mutants showed the most severe phenotype, especially the cruciferin triple mutant *crua crub cruc* and the napin RNAi line. Therefore, we further investigated the seed longevity mechanism provided by SSPs using these two mutants. SSPs were reported to be massively oxidized, especially in the form of carbonylation, during seed germination in *Arabidopsis* (Job *et al.*, 2005) and in pea (*Pisum sativum*) (Barba-Espín *et al.*, 2011). Different roles for SSPs in seed germination have been proposed due to its affinity to carbonylation: (i) Role in reserve mobilization. Carbonylated SSPs are easily destabilized from larger complexes, since they are more susceptible to proteolysis to remobilize resources for seed germination and seedling establishment. (ii) It was also suggested that the abundance of SSPs makes them an efficient scavenging system for ROS that are actively generated during seed germination (reviewed by El-Maarouf-Bouteau *et al.*, 2013). During long-term storage, SSPs are often carbonylated, which is an irreversible form of oxidation leading to deterioration, in dry aged seeds of *Arabidopsis* (Rajjou *et al.*, 2007; 2008) and beech (*Fagus sylvatica*) (Kalemba and Pukacka, 2014). The high abundance of SSPs might protect other proteins that are important for germination from oxidation, suggesting a role for SSPs in ROS-buffering during seed dry storage. We examined this hypothesis by investigation of the carbonylation pattern of AR and aged SSP mutant seeds in comparison with that of wild-type seeds.

**Fig. 6.**
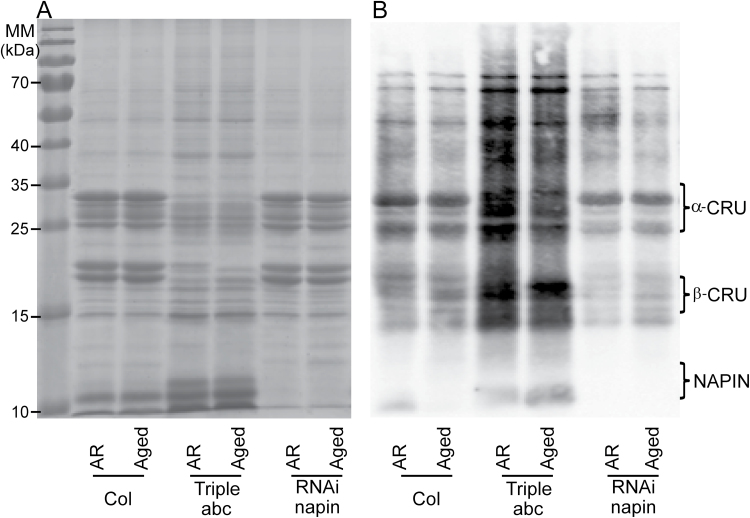
Protein carbonylation of seed proteins in after-ripened (AR) artificially aged seeds (Aged). (A) 1D gel electrophoresis stained with Coomassie Brilliant Blue of total seed protein extracts from Col, the triple cruciferin mutant abc (*crua crub cruc*) and the napin mutant (RNAi-napin). (B) Carbonylated proteins as detected by immunodetection of protein-bound DNP after derivatization with hydrazine.

1D-PAGE analysis of total protein extracts confirmed the reduction of cruciferin and napin proteins in dry seeds of the *crua crub cruc* and RNAi-napin mutant, respectively (Fig. 6A). Carbonylation of seed proteins was significant in both AR and aged seeds, with cruciferin being a major target in the wild-type and RNAi-napin lines (Fig. 6B). The cruciferin mutant exhibited a different protein carbonylation pattern in which carbonylation levels increased for the remaining proteins compared to the wild-type profile. In addition, there is a slight reduction in protein oxidation profiles comparing aged to AR seeds of all three genotypes, but also this effect was the strongest in the *crua crub cruc* mutant. Our result provides the first proof that SSPs, mainly cruciferins, are buffers for oxidative stress especially in dry seeds during storage. The carbonylated proteins in SSP mutants are interesting, since they will reveal new insights on the elements important for seed longevity.

## Conclusions

The naturally and artificially aged material used in this analysis allowed the molecular processes involved in seed ageing to be examined. In addition, the use of genetic material with different levels of seed longevity increased the likelihood that novel seed longevity factors would be identified. Despite being metabolically quiescent, the dry seed proteome was greatly altered upon ageing. Proteins that appear to be involved in seed longevity that were common to all genotypes included SSPs, proteins related to translation and energy metabolism (glycolytic and TCA pathways), and vitamin E synthesis (VTE1). These results indicated the importance of protection and maintenance of functional energetic and metabolic pathways, as well as antioxidant systems, for seed longevity. Interestingly, the different genotypes also expressed specific seed longevity pathways.

Our work presents the first evidence of ROS buffering by SSPs in dry seeds. This was revealed by analysing cruciferin double and triple mutants. We could not prove the participation of the other identified proteins in seed longevity, probably due to gene redundancy. Over-expression analyses of the identified genes or RNAi lines targeting whole gene families may be a better way to examine the effect of these proteins.

## Supplementary data

Supplementary data are available at *JXB* online.

Supplementary Fig. S1. Schematic presentation of the experiment.

Supplementary Fig. S2. Seed longevity of T-DNA insertion mutants after artificial ageing.

Supplementary Fig. S3. Schematic representation of energy metabolism-related pathways affected during seed storage.

Supplementary Table S1. The T-DNA insertion lines of the selected candidate genes.

Supplementary Table S2. Protein spots that show an overlapping abundance pattern in after-ripened seeds of NIL*GAAS1* and NIL*GAAS2* in comparison with that of L*er.*

Supplementary Table S3. The nomenclature of seed storage proteins.

Supplementary Data

## Abbreviations:

AR: after-ripening
CDT: controlled deterioration test
CRUs: cruciferins
GA: gibberellic acid
MW: molecular weight
pI: isoelectric point
PTM: post-translational modification
QTL: quantitative trait loci
ROS: reactive oxygen species
SSP: seed storage protein.
