# Supplementary material for: A role for seed storage proteins in Arabidopsis seed longevity
Source: J Exp Bot. 2015 Jul 16;66(20):6399–413. doi: 10.1093/jxb/erv348 (PMC4588887; doi:10.1093/jxb/erv348)
Supplement: Supplementary Data [file supp_66_20_6399__index.html]

A role for seed storage proteins in Arabidopsis seed longevity — Supplementary Data 

# A role for seed storage proteins in *Arabidopsis* seed longevity

## Supplementary Data

Data files

- Supplementary Data - Supplementary Data
