# Supplementary material for: A role for seed storage proteins in Arabidopsis seed longevity
Source: J Exp Bot. 2015 Jul 16;66(20):6399–413. doi: 10.1093/jxb/erv348 (PMC4588887; doi:10.1093/jxb/erv348)
Supplement: Supplementary Data [file supp_erv348_JEXPB151399_Supp_File__SH.pdf]

Supplemental figures and tables

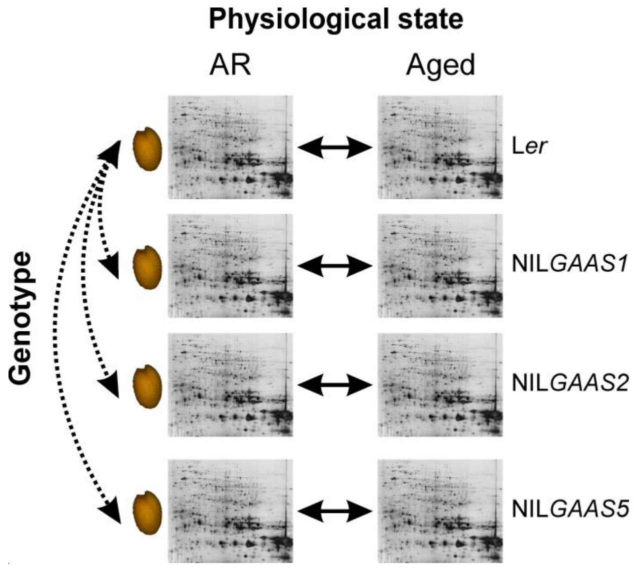

Figure S1. Schematic presentation of the performed experiment

Total soluble proteomes were isolated from dry seeds at two physiological states; after-ripened (AR) and aged and separated by 2D PAGE. Comparisons were made between the mentioned physiological states within the genotypes (indicated by the solid arrows); and between genotypes L*er* and near-isogenic lines; NIL*GAAS1*, NIL*GAAS2* and NIL*GAAS5* at the AR state (indicated by the dashed arrows).

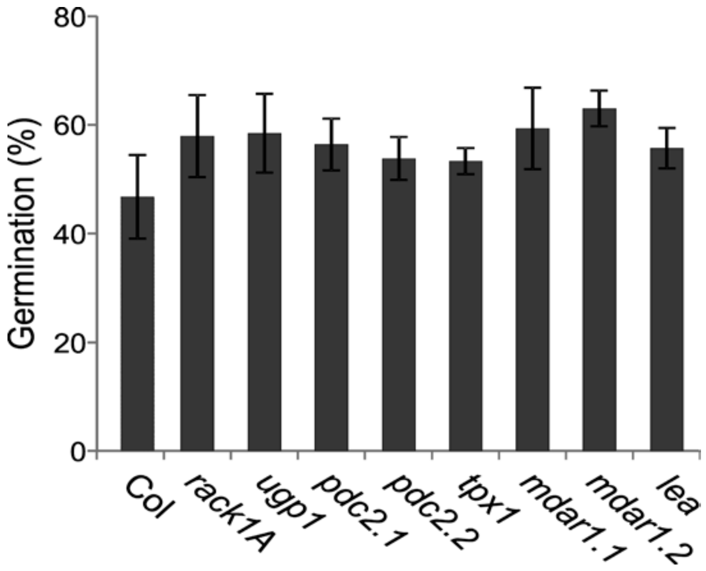

Figure S2. Seed longevity of T-DNA knock-out mutants after artificial aging

Seed longevity presented as germination (%) of T-DNA knock-out mutants of candidate genes was measured after 8 days of artificial aging treatment. Standard errors are calculated on four biological replicates. No significant differences between mutants and Col wild type were identified ($P \leq 0.05$).

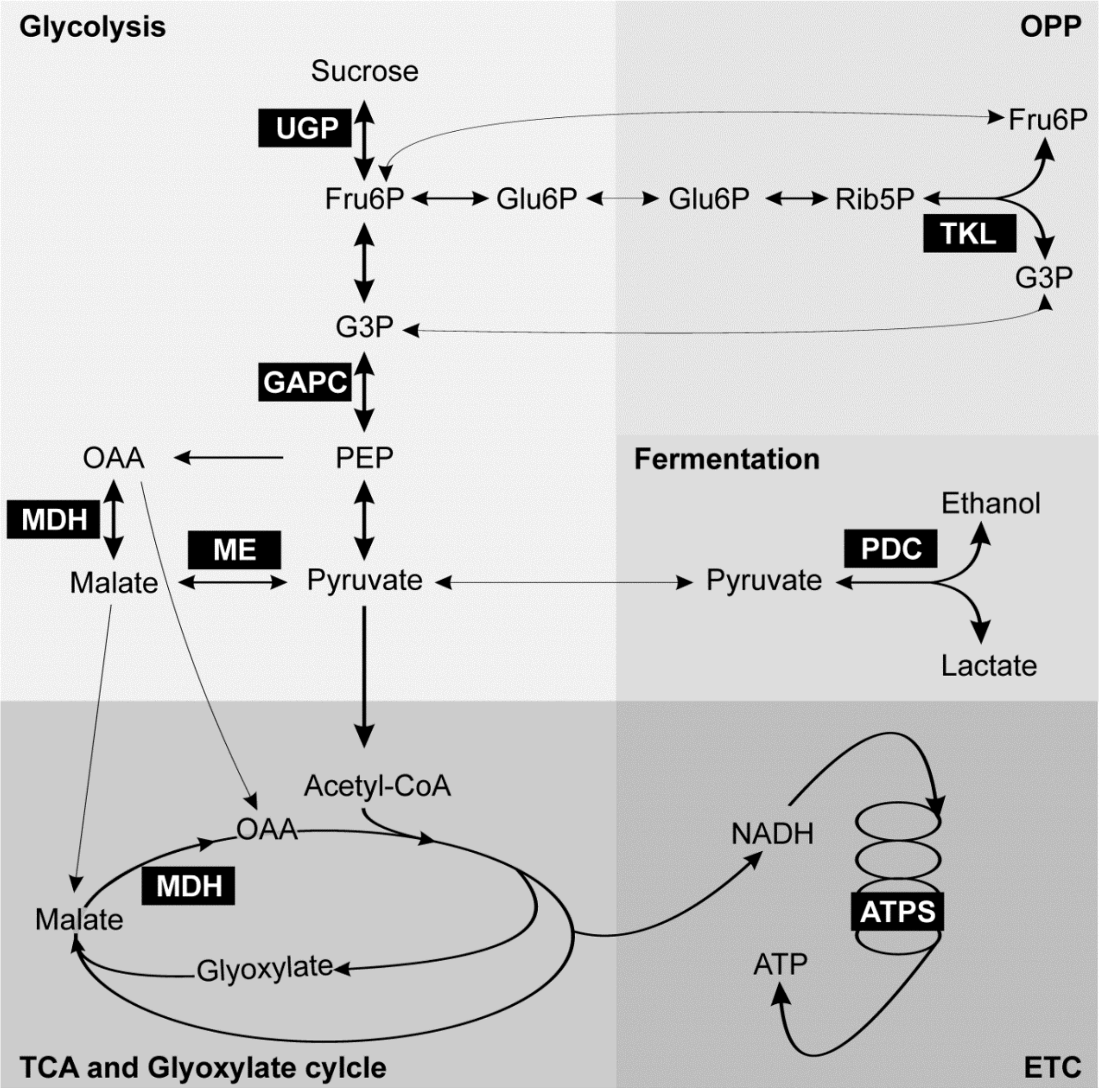

Figure S3. Schematic representation of energy metabolism related pathways affected during seed storage

Glycolysis, oxidative pentose phosphate (OPP), fermentation, tricarboxylic acid cycle (TCA), glyoxylate, and electron transport chain (ETC) pathways are shown. Identified enzymes are indicated in back box. UGP, UDP-1 GLUCOSE URIDYLYLTRANSFERASE; GAPC, GLYCERALDEHYDE3-P dehydrogenase C; TKL, TRANSKETOLASE; PDC, PYRUVATE DECARBOXYLASE; MDH, MALATE DEHYDROGENASE; ME, NADP-DEPENDENT MALIC ENZYME; ATPS, ATP SYNTHASE β chain; Fru6P, Fructose 6-phosphate; Glu6P, Glucose 6-phosphate; G3P, Glyceraldehyde 3-phosphate; PEP, Phosphoenolpyruvate; Rib5P, Ribulose 5-phosphate; OAA, Oxaloacetate.

Table S1. The T-DNA knock-out lines of the selected candidate genes

Details of the T-DNA knock-out lines that are tested for their role in seed longevity. For each gene the Gene ID and acronym, the T-DNA line code and the primer (left and right) information for confirming the insertion are presented in the knock-out. Homozygous T-DNA insertion lines were isolated for genes in bold.

Gene ID	Annotation	Acronym	T-DNA line	Insertion	Primer	
					Forward	Reverse
**AT3G52880**	Monodehydroascorbate reductase1	MDHAR 1	SALK_034893C	Exon	TTAGGTGGCTCCATATGAACG	ACCCCAACTATCACAATGTCG
		MDHAR 2	SALK_145224C	Exon	CTGAATTCCACTCTACGACTGG	ACACAGACCGATGCATTCTTC
**AT3G15670**	Late embryogeneis abundant protein	LEA76	SALK_052297.14.55	1000-Promotor	GGTCTCAAACGCTCAAAAATG	ACCATCGGGACATTTGTAGTG
**AT3G03250**	UTP:glucose-1-phosphate uridylyltransferase 1	UGP1	SALK_100183C	Exon	ATCATTTCATGGATGCTTTGC	AACCAGACATCACCAGACACC
AT5G17310	UTP:glucose-1-phosphate uridylyltransferase 2	UGP2	SALK_015923.25.50.	Exon	TCTTTGCTCCTTGCATGAAAC	GATTTCAAGTTTAGTCCCGGC
**AT5G54960**	Pyruvate decarboxylase 2	PDC2.1	SAIL_650_C05	Exon	TCCTGGTGATTTCAACCTGAC	CATGGCTTGAGCATAGCCTAG
		PDC2.2	SALK_009521C.	Exon	TTCAAGTTCAACCATATGGGC	TCACCAAGCTTAACCAAATCG
**AT1G65980**	Thioredoxin-dependent peroxidase1	TPX1	SALK_069621.55.70	300-UTR3	CTGATTTCTTCGTGAGCAAGC	AAGCATGGGGAAAAACATACC
**AT1G18080**	Receptor for activated kinase1	RACK1A	SALK_009406C		Provided by Magdalena Gamm	
AT2G15430	RNA polymerase II		SALK_008128.30.20.	Exon	ATGGATCCAGTCACCAGACAG	CGTTTATTTTCGTTACGAGCG
AT5G28840	GDP-D-mannose 3´,5´epimerase		SALK_018413.54.75	Exon	TTTTTCGGATCATCTCGAGTC	TCGATGAGTGTGTTGAAGGTG
AT5G28840	GDP-D-mannose 3´,5´epimerase	GME	SALK_150208.40.70	Exon	TCACCGTGAGTGATCCCTAAC	TGCTTGGATCAACTTAGGTGG

Table S2. Protein spots that show an overlapping abundance pattern in after-ripened seeds of NIL*GAAS1* and NIL*GAAS2* in comparison with that of L*er*

The table displays protein spots based on the seven comparisons. Spot ID, the gene corresponding to the protein underlying the spot, molecular weight (MW in kD) and the theoretical (Th) and experimental (Exp) isoelectric point (pI), are presented respectively. Furthermore the relative abundance (fold change) of the spots in both types of comparisons (Fig. 1; physiological state and genotype) is indicated. Positive fold changes indicate higher abundances, and negative lower abundances. Fold changes in bold indicate the is statistically significant changes. N*G1* stands for NIL*GAAS1*, N*G2* for NIL*GAAS2* and N*G5* for NIL*GAAS5*. Spots that were identified based on comparison to the reference protein map (http://www.seed-proteome.com) are labelled with [R]. The n.i.. indicates not idenitified.

			MW (kD)		pI		Relative abundance (fold change)						
Spot ID	Gene	Protein	Th	Exp	Th	Exp	Physiological state: Aged vs. AR				Genotype AR: NIL vs. L*er*		
							L*er*	N*G1*	N*G2*	N*G5*	N*G1*	N*G2*	N*G5*
**ID0678**	n.i.	n.i.	n.i..	51.13	n.i..	6.84	1.4	**-1.9**	**-2.1**	1.3	**-1.9**	**-2.1**	1.3
eID0179	AT4G28520	Cruciferin C	58.24	27.37	6.99	5.71	1.3	**1.8**	**1.5**	1.2	**1.8**	**1.5**	1.2
	AT1G03880	Cruciferin B	50.56		7.0								
	AT1G03890	Cupin family protein	49.67		5.45								
	AT5G07190	Arabidopsis thaliana seed gene 3	23.08		6.74								
ID0807	AT4G09670	Oxidoreductase family protein	39.56	41.25	5.69	5.69	1.1	**1.6**	**1.6**	1.5	**1.6**	**1.6**	1.5
eID0141 [R]	AT5G44120	Cruciferin A	52.59	14.51	7.88	5.86	**2.5**	**1.5**	**1.7**	1.6	**1.5**	**1.7**	1.6

Table S3. The nomenclature of seed storage proteins

Single, double and triple knock-outs lines for cruciferins and RNAi line for napins obtained from Withana-Gamage *et al.* (2013).

Line	Acronym	Knock-out gene	Protein content
Col	Col	-	CRUA, CRUB, CRUC and NAPINs
Single	**a**BC	AT5G44120	CRUB, CRUC and NAPINs
	A**b**C	AT1G03880	CRUA, CRUC and NAPINs
	AB**c**	AT4G28520	CRUA, CRUB and NAPINs
Double	**ab**C	AT5G44120 and AT1G03880	CRUC and NAPINs
	**a**B**c**	AT5G44120 and AT4G28520	CRUB and NAPINs
	A**bc**	AT1G03880 and AT4G28520	CRUA and NAPINs
Triple	**abc**	AT5G44120, AT1G03880 and AT4G28520	CRU- and NAPINs
RNAi	RNAi Napin	Napin gene family (5 members)	CRUs
